# Intratracheal inoculation of AHc vaccine induces protection against aerosolized botulinum neurotoxin A challenge in mice

**DOI:** 10.1038/s41541-021-00349-w

**Published:** 2021-06-22

**Authors:** Changjiao Gan, Wenbo Luo, Yunzhou Yu, Zhouguang Jiao, Sha Li, Duo Su, Junxia Feng, Xiaodong Zhao, Yefeng Qiu, Lingfei Hu, Dongsheng Zhou, Xiaolu Xiong, Jinglin Wang, Huiying Yang

**Affiliations:** 1 State Key Laboratory of Pathogen and Biosecurity, Beijing Institute of Microbiology and Epidemiology, Beijing, China; 2 Department of Protein Engineering, Beijing Institute of Biotechnology, Beijing, China; 3 Laboratory Animal Center, Academy of Military Medical Science, Beijing, China

**Keywords:** Immunology, Microbiology

## Abstract

Botulinum neurotoxin (BoNT), produced by *Clostridium botulinum*, is generally known to be the most poisonous of all biological toxins. In this study, we evaluate the protection conferred by intratracheal (i.t.) inoculation immunization with recombinant Hc subunit (AHc) vaccines against aerosolized BoNT/A intoxication. Three AHc vaccine formulations, i.e., conventional liquid, dry powder produced by spray freeze drying, and AHc dry powder reconstituted in water are prepared, and mice are immunized via i.t. inoculation or subcutaneous (s.c.) injection. Compared with s.c.-AHc-immunized mice, i.t.-AHc-immunized mice exhibit a slightly stronger protection against a challenge with 30,000× LD_50_ aerosolized BoNT/A. Of note, only i.t.-AHc induces a significantly higher level of toxin-neutralizing mucosal secretory IgA (SIgA) production in the bronchoalveolar lavage of mice. In conclusion, our study demonstrates that the immune protection conferred by the three formulations of AHc is comparable, while i.t. immunization of AHc is superior to s.c. immunization against aerosolized BoNT/A intoxication.

## Introduction

Botulinum neurotoxin (BoNT), which is secreted by *Clostridium botulinum*, is the most poisonous substance known in nature^1^. It is classified into eight serotypes (A–H)^2,3^, with sequence differences of 37.2–69.6% at the amino acid level^4^. BoNT/A is the most toxic for humans^5,6^. BoNT exerts its pathological effects by inhibition of acetylcholine release through binding to peripheral cholinergic nerve endings, leading to flaccid paralysis and ultimately death^5,7–10^. BoNT can be disseminated by food, water, or air, and enter the body through mucosal surfaces. It is most likely to be disseminated by bioterrorists through air. It can lead to neuronal tissue damage, so it requires extraordinary biosafety precautions^9,11^.

Vaccination leads to the generation of neutralizing antibodies against BoNT, which is the most effective method for preventing BoNT intoxication. A pentavalent botulinum ABCDE toxoid vaccine was administered to military personnel and workers at risk of exposure to BoNT. However, its disadvantages include high production costs, it is time consuming to produce, and it is dangerous during detoxification^12^. The BoNT Hc subunit vaccines, employing purified protein antigens rather than intact toxin, have been demonstrated to be highly effective vaccines, providing complete protection against the parental toxin^13–16^. However, subunit vaccines usually require the addition of adjuvants for enhanced immunogenicity, where a balance must be found between strong activation of the immune system and low toxicity^17^. Several recombinant Hc vaccine candidates for BoNT serotypes A–F have been developed^7,18–20^, and alternative administration routes have been explored in animal models, including inhalation and ingestion^21–23^.

Mucosa is the first line of defense against inhaled toxins and microbes. Vaccines administered by subcutaneous and intramuscular injection usually evoke a systemic immune response without a mucosal IgA response in the respiratory tract^8,21,24,25^. Moreover, they need to be administered by trained personnel and transported via an expensive cold chain. Pulmonary vaccination has many advantages over conventional vaccination routes. The pulmonary system has a large surface area for absorption, and pulmonary vaccination is non-invasive^26^ and can induce long-lasting systemic and mucosal immune responses against respiratory pathogens and toxins^21,27–29^. Therefore, inhalable recombinant Hc subunit (AHc) dry powder for pulmonary vaccination offers an interesting strategy^30^. To protect people from neurotoxicity induced by inhaled BoNT/A, which can be used in bioterrorism, in the present study, we explored whether pulmonary vaccination with AHc could induce specific mucosal and systemic immune responses, and thereby improve the protection against aerosolized BoNT. Using a mouse model of delivery through intratracheal (i.t.) inoculation^29,31^, we found that i.t. immunization of AHc vaccines in three different formulations (liquid, dry powder, and reconstituted powder) could induce protection against 30,000× LD_50_ of BoNT/A aerosol challenge in BALB/c mice, which is associated with the production of BoNT/A-neutralizing AHc-specific SIgA.

## Results

### Preparation and characterization of inhalable AHc vaccines

We prepared three different formulations of AHc vaccines for i.t. immunization, i.e., liquid, dry powder, and powder reconstituted in water. Briefly, AHc was dissolved in PBS mixed with CpG oligodeoxynucleotide (CpG) as a mucosal adjuvant^32–34^ to obtain the AHc liquid vaccine. AHc was dissolved in a solution containing excipients (d-Mannitol, myo-inositol, l-Leucine, and poloxamer 188) and CpG (Table 1) and spray dried to obtain AHc dry powder. The AHc dry powder was reconstituted in water at a ratio of 10:1 (w/v) to obtain the reconstituted AHc powder vaccine.

**Table 1:** Composition and proportion of AHc spray-drying solution.

Composition	Proportion	Properties	Supplier	References
CpG	0.1% (w/v)	Adjuvant	Invitrogen, USA	^32–34^
d-Mannitol	1% (w/v)	Increases dispersibility	Sigma-Aldrich, Shanghai, China.	^47,61–63^
Myo-inositol	1% (w/v)	Enhances stability		^64,65^
l-Leucine	0.5% (w/v)	Enhances dispersibility and stability		^54,62,66^
Poloxamer 188	0.05% (v/v)	Enhances stability, surfactants		^11,56^

**Fig. 1:**
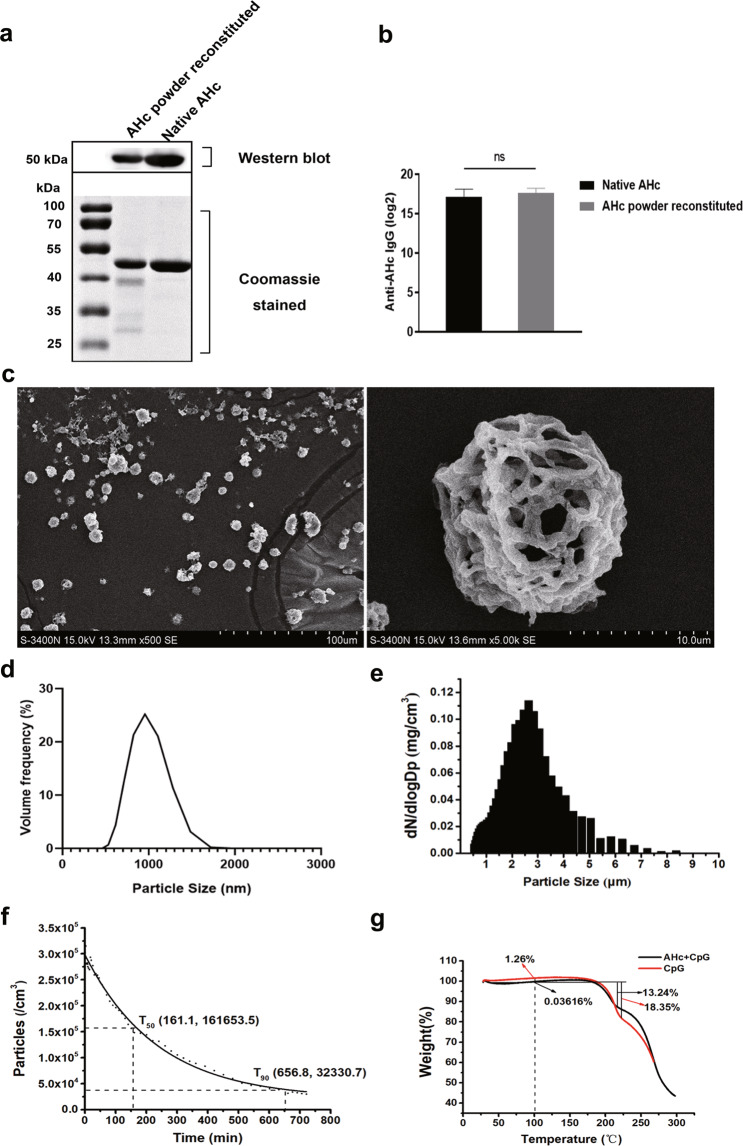
Characterization of AHc dry powder. **a** AHc dry powder and AHc liquid were analyzed by western blot using mouse polyclonal antibodies to AHc (upper panel). A protein gel stained with Coomassie brilliant blue was used as a loading control (lower panel). **b** The immunogenicity of AHc dry powder and AHc liquid was analyzed by ELISA using mouse polyclonal antibodies to AHc. **c** Scanning electron microscopy images of AHc dry powder. **d** The Z-average of the mean size of the AHc dry powder, as determined using a Malvern Zetasizer Nano S90. **e** The MMAD of AHc aerosol dry powder, as measured using a TSI Model 3321 Aerodynamic Particle Sizer (APS) aerosol spectrometer (TSI APS spectrometer 3321). **f** Sedimentation of AHc dry powder, as measured using a TSI APS spectrometer 3321. **g** TGA of the AHc dry powder and CpG dry power (control).

For characterization of the AHc dry powder, the structural integrity and antigenicity of AHc after spray freeze drying (SFD) powder were analyzed. As shown in Fig. 1a and Supplementary Fig. 1, the molecular weight of reconstituted AHc powder was identical to that of the liquid formulation (~50 kDa), indicating that the potency of AHc was not affected by the SFD process. As shown in Fig. 1a, b, both reconstituted powder and the liquid formulation reacted with mouse polyclonal antibodies to AHc, and the titers were not significantly different. These results demonstrated that the SFD process did not affect the biochemical integrity^35^ and the immunogenicity of AHc.

Scanning electron micrographs (Fig. 1c) revealed that the AHc dry powder consisted of particles that were spherical and porous. The geometrical mean particle size of the subunit vaccine powder, as determined by laser diffraction analysis, was in the range of 0.5–3.0 µm (Fig. 1d). Moreover, the mass median aerodynamic diameter (MMAD) of AHc aerosol particles, as measured by an aerodynamic particle sizer (APS) spectrometer 3321, was 2.26 ± 0.25 µm (Fig. 1e). The curve of total aerosol AHc particles was shown in Fig. 1f, which reflects the suspension time of AHc dry powder aerosol in the air. The fitting function formula *y* = 21049.9 + 277513.7 × exp(−*x*/236.9) was used to calculate that the aerosol concentration decreased to 50% of the initial value after about *T*_50_ = 161.1 min, and the aerosol concentration decreased to 10% of the initial value after about *T*_90_ = 656.8 min. The residual moisture content in the AHc dry powders was 0.036% w/w, as determined by thermogravimetric analysis (TGA) (Fig. 1g).

### Systemic humoral and lung mucosal immune responses induced by intratracheal inoculation of AHc vaccines

**Fig. 2:**
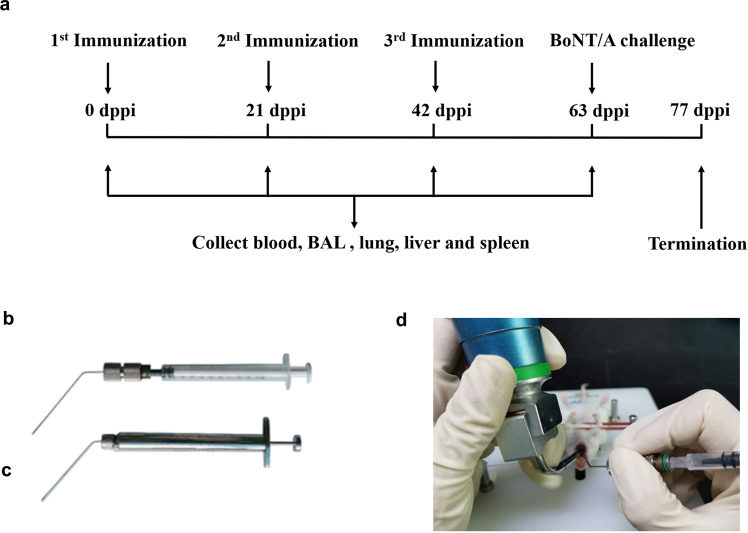
Schematic of immunization via intratracheal inoculation method. **a** The immunization scheme, **b** powder MicroSprayer Aerosolizer (Huironghe Company, Beijing, China), and **c** liquid MicroSprayer Aerosolizer (Huironghe Company, Beijing, China) used for i.t. inoculation. **d** Representative image of non-invasive i.t. powder inoculation. Anesthetized mice were placed on a slanted board. The trachea was exposed using a laryngoscope, the MicroSprayer Aerosolizer was inserted 25 mm from the larynx, and the powder was sprayed. BAL bronchoalveolar lavage, dppi days post primary immunization.

To evaluate the protection of i.t. immunization with AHc vaccines against aerosolized BoNT/A intoxication, the three AHc vaccine formulations were used to immunize mice via the i.t. or the s.c. route for three times at 3-week intervals^29,36^ as previously described. The immunization scheme and groups are shown in Fig. 2a and Table 2, respectively. The i.t. immunization was performed using a laryngoscope and a powder MicroSprayer Aerosolizer (Fig. 2b) or a liquid MicroSprayer Aerosolizer (Fig. 2c) according to a previously described method (Fig. 2d)^29,31^.

**Table 2:** Summary of all immunization groups in the experiment.

Immunization route	Group	Formulation type	AHc dose (µg/mouse)	CpG dose (µg/mouse)	Volume (µL/mouse)
i.t.	AHc	Liquid	20	20	50
i.t.	AHc	Powder	20	20	50
i.t.	AHc	Powder reconstituted	20	20	50
i.t.	CpG	Liquid	/	20	50
i.t.	CpG	Powder	/	20	50
i.t.	CpG	Powder reconstituted	/	20	50
i.t.	PBS	Liquid	/		50
s.c.	AHc	Liquid	20	20	100
s.c.	AHc	Powder reconstituted	20	20	100
s.c.	CpG	Liquid	/	20	100
s.c.	CpG	Powder reconstituted	/	20	100
s.c.	PBS	Liquid	/		100

**Fig. 3:**
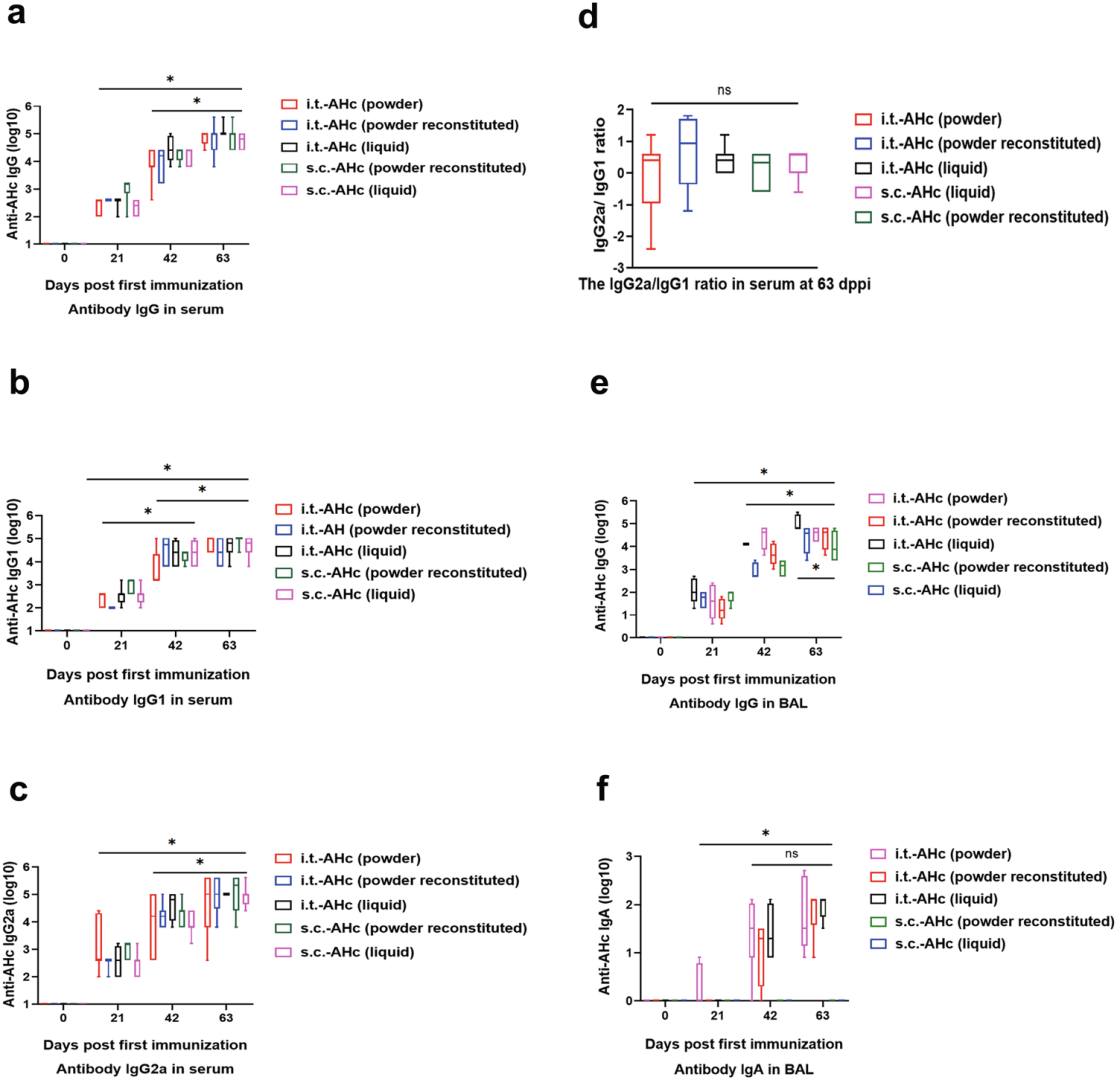
Antibody responses to AHc, as determined by ELISA. Serum was collected from eight mice from each of the following 12 groups: (1) i.t.-AHc (powder), (2) i.t.-AHc (powder reconstituted), (3) i.t.-AHc (liquid), (4) i.t.-CpG (powder), (5) i.t.-CpG (powder reconstituted), (6) i.t.-CpG (liquid), (7) s.c.-AHc (powder reconstituted), (8) s.c.-AHc (liquid), (9) s.c.-CpG (powder reconstituted), (10) s.c.-CpG (liquid), (11) i.t.-PBS, or (12) s.c.-PBS, at 0, 21, and 42 dppi. Moreover, BAL was collected from four mice per group at 0, 21, and 42 dppi. **a** The reciprocal titers of IgG to AHc in mouse sera. **b** The reciprocal titers of IgG1 to AHc in mouse sera. **c** The reciprocal titers of IgG2a to AHc in mouse sera. **d** The IgG2a/IgG1 ratio in serum at 63 dppi. **e** The reciprocal titers of IgG to AHc in mouse BAL. **f** The reciprocal titers of SIgA to AHc in mouse BAL. The antibody titers are presented as mean ± SEM of eight serum samples or four BAL samples per group. Statistical differences were calculated by two-way ANOVA, followed by least significant difference (LSD) analysis or Tukey’s test. *P < 0.05.

To compare the humoral immune responses between mice immunized by different routes (i.t. vs. s.c.) and different AHc formulations (liquid, powder, and reconstituted powder), the titers of AHc-specific IgG, IgG1, and IgG2a in mouse sera were determined by ELISA at 21 days post primary immunization (dppi). As a result, the AHc-specific IgG, IgG1, and IgG2a levels in serum of i.t.-AHc-immunized mice were not significantly different from those in serum of s.c.-AHc-immunized mice at 21, 42, or 63 dppi, and no significant difference was observed among the different vaccine formulations (*P* > 0.05, Fig. 3a–c). The levels of AHc-specific IgG, IgG1, and IgG2a in i.t.-AHc- or s.c.-AHc-immunized mice at 63 dppi were significantly higher those at 21 and 42 dppi (*P* < 0.01, Fig. 3a–c). All immunized mice exhibited a similar IgG2a/IgG1 ratio, indicating a balanced Th1/Th2 response (Fig. 3d).

To evaluate whether the i.t.-AHc immunization induced a mucosal immune response, levels of AHc-specific IgG and SIgA in bronchoalveolar lavage (BAL) of i.t.-AHc and s.c.-AHc-immunized mice were measured by ELISA (Fig. 3e, f). AHc-specific IgG was detected; the levels in the i.t.-AHc- and s.c.-AHc-immunized groups were significantly higher at 63 dppi than at 21 and 42 dppi (*P* < 0.05, Fig. 3e), and no significant difference in AHc-specific IgG levels in BAL was observed between different vaccine formulations (*P* > 0.05, Fig. 3e).

Interestingly, i.t.-AHc immunization induced significantly higher levels of AHc-specific SIgA in BAL of mice at 21, 42, and 63 dppi than s.c.-AHc immunization (*P* < 0.05, Fig. 3f). AHc-specific SIgA was detected in BAL after immunization with any of the three formulations in i.t.-AHc mice as early as 21 dppi, and the levels increased gradually during the immunization course (*P* < 0.05, Fig. 3f). No significant difference in AHc-specific IgA levels in BAL was observed between different vaccine formulations in i.t.-AHc-immunized mice (*P* > 0.05, Fig. 3f).

### Cellular immune response induced by intratracheal inoculation of AHc

To compare the T cell immune response induced by different routes or different vaccine formulations, splenic cells were isolated from immunized mice and IFN-γ and interleukin (IL)-4 levels were determined by enzyme-linked immunospot (ELISPOT) analysis. As shown in Supplementary Fig. 2, the levels of IFN-γ in i.t.-AHc-immunized mice were not significantly different from those in s.c.-AHc-immunized mice, but they were both significantly higher than in the control group (*P* < 0.05). However, we failed to detect any significant difference in IL-4 secretion in AHc-immunized mice compared with control mice (data not shown).

### Enhanced protection induced by i.t.-AHc immunization against aerosolized BoNT/A

**Fig. 4:**
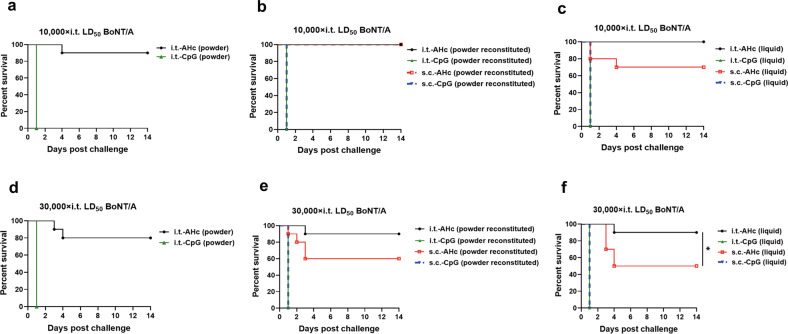
Protection against a lethal dose of BoNT/A aerosol induced by immunization with AHc vaccines via i.t. inoculation or s.c. injection. Mice were immunized by i.t. inoculation or s.c. injection with AHc vaccines or CpG adjuvants in three formulations three times at 21-day intervals. **a**, **d** Dry powder formulation. **b**, **e** Reconstituted powder formulation. **c**, **f** Liquid formulation. Then mice were challenged with 10,000× i.t. LD_50_ (**a**–**c**) or 30,000× i.t. LD_50_ (**d**–**f**) of BoNT/A in 50 µl of PBS via the i.t. route. Each group consisted of ten mice. The animal survival rate was analyzed by Kaplan–Meier survival analysis. *P < 0.05. Data shown were from one representative experiment with two technical replicates.

In our mouse model of BoNT/A inhalation, the LD_50_ of i.t. BoNT/A challenge was 67.45 ng/kg, which is about five times higher than that of s.c. challenge (12.34 ng/kg, Supplementary Fig. 3). To compare the immune protection against high-dose BoNT/A aerosol challenge conferred by different vaccination routes and formulations, all mice were i.t. challenged with a dose of 10,000× LD_50_ BoNT/A at 63 dppi. All PBS-immunized mice (data not shown) and CpG-immunized mice (Fig. 4a–c) died within 1 day. One mouse from the i.t.-AHc (powder)-immunized group and three mice from the s.c.-AHc (liquid)-immunized group died within 4 days (Fig. 4a, c). All mice in the other groups survived until the end of the experiment (14 days post challenge) (Fig. 4b, c). The survivors began to gain weight after day 4 postchallenge (Supplementary Fig. 4a). The survival percentage of mice in each of the AHc-immunized groups after 14 days was over 70%, which was significantly higher than that of CpG-immunized and PBS-immunized mice (*P* < 0.05). No significant difference with respect to the protection conferred by i.t. and s.c. vaccination (*P* > 0.05) or with respect to the protection conferred by liquid, dry powder, or reconstituted powder formulations was observed (*P* > 0.05).

When mice were challenged with a higher dose of 30,000× i.t. LD_50_ BoNT/A at 63 dppi (ten mice/group), all PBS-immunized mice (data not shown) and CpG-immunized mice (Fig. 4d–f) died within 1 day. Five mice of the s.c.-AHc (liquid)-immunized group and four mice of the s.c.-AHc (powder reconstituted)-immunized group died within 4 days (Fig. 4e, f), and two mice of the i.t.-AHc (powder)-immunized group, one mouse of the i.t.-AHc (liquid)-immunized group, and one mouse of the i.t.-AHc (powder reconstituted)-immunized group died within 4 days (Fig. 4d–f). The survivors from all groups began to gain weight after day 4 postchallenge (Supplementary Fig. 4b). The survival percentage of the i.t.-AHc (powder reconstituted)-immunized group was slightly higher than that of the s.c.-AHc (powder reconstituted)-immunized group (Fig. 4e), while the survival percentage of the i.t.-AHc (liquid)-immunized group was significantly higher than that of the s.c.-AHc (liquid)-immunized group (*P* < 0.05, Fig. 4f). In addition, no significant difference was observed in the survival percentage of mice between the liquid, dry powder, and reconstituted powder AHc-immunized groups (*P* > 0.05).

### Lung mucosal SIgA levels after i.t. immunization with AHc-neutralized BoNT/A in mice

**Fig. 5:**
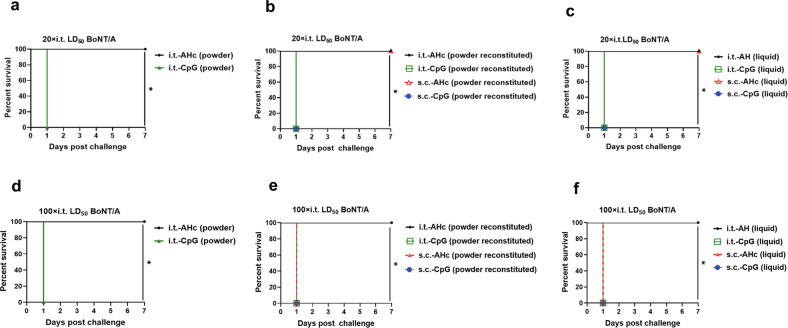
BAL from AHc-immunized mice neutralized the toxicity of 20× i.t. LD_50_ or 100× i.t. LD_50_ BoNT/A before challenge via the i.t. route. BoNT/A was incubated with BAL from AHc-immunized mice in PBS (1:1) for 30 min at room temperature. **a**, **d** BAL from dry powder formulation-immunized mice. **b**, **e** BAL from reconstituted powder formulation-immunized mice. **c**, **f** BAL from liquid formulation-immunized mice. Then mice were challenged with **a**–**c** 20× i.t. LD_50_ (*n* = 5) or **d**–**f** 100× i.t. LD_50_ (*n* = 10) of BoNT/A that had been preincubated with BAL from different groups. The animal survival rate was analyzed by Kaplan–Meier survival estimates. *P < 0.05.

Both i.t. and s.c. vaccination could induce significant increases in the levels of AHc-specific IgG, while only i.t. vaccination induced an AHc-specific SIgA response in mucosal sites. Therefore, we determined whether these AHc-specific SIgA also provided protection against mucosal BoNT/A intoxication. Mice were i.t. challenged with 20× i.t. LD_50_ or 100× i.t. LD_50_ of BoNT/A that had been preincubated with BAL from different groups. As results, all mice challenged with 20× i.t. LD_50_ or 100× i.t. LD_50_ of BoNT/A that was preincubated with BAL from i.t.-AHc-immunized survived until the end of the experiment, while mice challenged with 20× i.t. LD_50_ but not 100× i.t. LD_50_ of BoNT/A that was preincubated with BAL from s.c.-AHc-immunized mice survived until the end of the experiment (Fig. 5a–f). All mice challenged with 20× i.t. LD_50_ or 100× i.t. LD_50_ of BoNT/A that was preincubated with BAL from CpG-immunized mice (Fig. 5a–f) and PBS-immunized mice (data not shown) died within 1 day. These results indicate that both AHc-specific IgG and SIgA in the BAL could neutralize the toxicity of BoNT/A, while AHc-specific SIgA alone might contribute to the enhanced neutralizing activity.

**Fig. 6:**
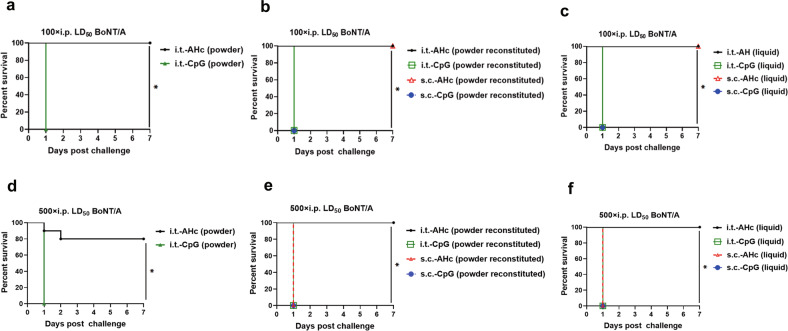
BAL from i.t.-AHc-immunized mice neutralized the toxicity of 100× i.p. LD_50_ or 500× i.p. LD_50_ before challenge via the i.p. route. BoNT/A was incubated with BAL from AHc-immunized mice in PBS (1:1) for 30 min at room temperature. **a**, **d** BAL from dry powder formulation-immunized mice. **b**, **e** BAL from reconstituted powder formulation-immunized mice. **c**, **f** BAL from liquid formulation-immunized mice. Then mice were challenged with **a**–**c** 100× i.p. LD_50_ (*n* = 5) or **d**–**f** 500× i.p. LD_50_ (*n* = 10) of BoNT/A that had been preincubated with BAL from different groups. The animal survival rate was analyzed by Kaplan–Meier survival estimates. *P < 0.05.

These results prompted us to explore whether AHc-specific SIgA in BAL from i.t.-AHc-immunized groups could protect mice against BoNT/A challenge via other routes, such as i.p. injection. In this effort, mice were i.p. challenged with 100× i.p. LD_50_ or 500× i.p. LD_50_ of BoNT/A that had been preincubated with BAL from different groups. As expected, all mice challenged with 100× i.p. LD_50_ or 500× i.p. LD_50_ of BoNT/A that was preincubated with BAL from i.t.-AHc-immunized mice survived until the end of the experiment, while mice challenged with 100× i.p. LD_50_ but not 500× i.p. LD_50_ of BoNT/A preincubated with BAL from s.c.-AHc-immunized mice survived until the end of the experiment (Fig. 6a–f). And all mice challenged with BoNT/A that was preincubated with BAL from CpG-immunized mice (Fig. 6a–f) and PBS-immunized mice (data not shown) died within 1 day. These results indicated that AHc-specific SIgA in BAL may also play an important role in the enhanced protection against BoNT/A challenge via the i.p. route.

### Intratracheal inoculation of AHc caused no detectable pathological lesions in mouse organs

To evaluate the safety of i.t. immunization of AHc, four mice per group were euthanized and part of their lung, spleen, and liver was collected 21 days after each immunization. Each organ was fixed for pathological analysis by light microscopy. As shown in Supplementary Fig. 5a–c, no obvious pathological lesion was observed in lungs, livers, and spleens of i.t.-AHc- or s.c.-AHc-immunized mice. In addition, the pathological scores revealed no significant difference between the CpG-, PBS-, and AHc-immunized groups, confirming the safety of i.t.-AHc immunization (Supplementary Fig. 5d–f).

## Discussion

Since exposure to aerosols contaminated by BoNT is considered a potential way of taking up BoNT, the development of an effective and safe mucosal vaccine against this toxin is of high priority^37^. Some reports have investigated the role of SIgA responses in BoNT intoxication^8,21^. In the present study, we have established a mouse model of i.t. delivery of AHc to investigate the mucosal and humoral immune protection against inhalational BoNT intoxication. Using this model, we demonstrated that i.t.-AHc immunization could induce not only systemic humoral and cell-mediated immune responses, but also strong mucosal IgA and IgG responses in the lungs. We also demonstrated that i.t.-AHc- and s.c.-AHc-immunized mice were protected to BoNT/A aerosol challenges as high as 30,000× LD_50_ of BoNT/A, while the survival percentage of i.t.-AHc-immunized mice was slightly higher than that of s.c.-AHc-immunized mice. There results indicate that i.t. immunization is superior to s.c. immunization against pulmonary BoNT intoxication.

IgA is the predominant antibody isotype produced at mucosal surfaces^38^, and the second most abundant antibody isotype in the circulation. SIgAs are exclusively present at mucosal surfaces^39^ and have superior neutralization activity^40^. Specific SIgAs can neutralize pathogens and prevent them from invading through the mucosal epithelium, which has been reported for other toxins as well^41,42^. TcdA_26–39_-specific IgA could prevent *Clostridioides difficile* vegetative cells from attaching to the mucosal epithelium at the early stage, which might be a key step in the infection process^43^. Intranasal vaccination of an adenoviral vector Ad/opt-BoNT/C-H_C_50^44^ or a BoNT/A toxoid plus a cholera toxin mutant (E112K) could elicit elevated SIgA levels and used as nasal vaccines to prevent BoNT intoxication^8^. In the present study, mice challenged via either the i.p. or the i.t. route with high dose of BoNT/A that was preincubated with BAL from i.t.-AHc-immunized mice survived, suggesting that AHc-specific SIgA in the BAL might contribute to the enhanced neutralizing activity and prevent BoNT/A intoxication through different routes. However, additional experiments need to be performed to confirm the functional and essential roles of AHc-specific SIgA antibodies in the enhanced protection against BoNT/A, such as evaluating the efficacy of recombinant AHc subunit vaccine via i.t. inoculation in WT and IgA-deficient (IgA (−/−)) mice^8^.

Pulmonary delivery of dry powder has become increasingly attractive in recent years^45,46^. In this study, the SFD technology was used to prepare AHc dry powder, which could effectively reduce the crystallization of formulation ingredients, enhance the amorphous glassy state and protein activity of powder formulation than that prepared by spray drying^35,47,48^. Then the protection efficacy of AHc dry powder was compared with that of AHc liquid vaccine. Our results demonstrated that i.t. delivery of a dry powder formulation of AHc confers protection against a lethal aerosolized BoNT challenge that is at least as strong as the protection conferred by i.t. delivery of a liquid formulation. Moreover, there are several potential advantages of dry powder over liquid vaccines. First, the addition of excipients could increase the total amount of dry powder used, which is beneficial for the administration of high quantities. Second, the powder formulation may have increased storage stability and reduced transportation costs, which is becoming even more important for the development of vaccines. Finally, effective mucosal immunization could improve the bioavailability of powder^49^. The present study implies that the AHc dry powder vaccine containing the mucosal adjuvant CpG prepared by SFD with i.t. inoculation is feasible, and that it could be developed as an effective alternative to improve the existing BoNT vaccination.

In summary, we demonstrated that i.t. immunization with an SFD AHc dry powder vaccine with i.t. inoculation was feasible, which could increase storage stability and reduced transportation costs of AHc vaccines. However, compared to liquid formulation and reconstituted powder formulation, i.t. immunization of AHc dry powder was not the most suitable for nebulization. To achieve the optimum immune efficacy, the AHc could be prepared into dry powder formulation by SFD for storage stability increasing as well as transportation costs reducing, and be dispersed in liquid before i.t. immunization.

## Methods

### Animals and ethics statement

Six-week-old female BALB/c mice (SPF) were obtained from Charles River Laboratories (Beijing, China). All infectious experiments were conducted in the animal biosafety level-3 laboratory. This study was performed with the permission of Institute of Animal Care and Use Committee (IACUC) at the Academy of Military Medical Science, and the ethical approval number was IACUC-DWZX-2020-049.

### Preparation of BoNT/A

The BoNT/A complex (including type A neurotoxin and six or seven nontoxic proteins^6^) was isolated from the bacterial *C. botulinum* type A strain 62A, based on a modified version of a previously described extraction method^50,51^. Bacteria were cultured in PYG medium for 4 days, and the protein complex was isolated from the bacterial culture by acid precipitation, ion-exchange chromatography, and gel filtration. The BoNT/A complex was stored at −80 °C until use.

### Expression and purification of AHc

The pTIG-Trx-Hc plasmid, which expresses recombinant AHc, was kindly provided by Dr. Yu from the Beijing Institute of Biotechnology as a generous gift. AHc was expressed in *E. coli* strain BL21 (DE3) (Agilent Technologies, Santa Clara, CA, USA) and purified by affinity chromatography as described previously^52^. The purified AHc was analyzed by SDS-PAGE and western blot using hyperimmune horse toxin A antiserum (from the National Institute for the Control of Pharmaceutical and Biological Products, Beijing, China). The gels and blots that use the same reagents and that are presented in the same panel derive from the same experiment and were processed in parallel.

### Preparation of different formulations of the AHc vaccine

AHc was dissolved in spray drying solution containing D-Mannitol, myo-inositol, l-Leucine, and poloxamer 188 as excipients and CpG as a mucosal adjuvant for SFD (Table 1). We had investigated and optimized the different combinations of excipients mentioned above, with trypsinogen as a model protein by spray-drying in previous studies (unpublished data). Overall, 20–40 mL of a solution with a total solute concentration of 2.75% (w/v) was passed through a two-fluid pneumatic spray nozzle with a diameter of 2 mm (TSE Inc, Germany) at a liquid feed rate of 5 mL/min. The two-fluid pneumatic spray nozzle was located at a distance of 10 cm above the stainless-steel container. The liquid was forced into small droplets with a fixed back pressure of 1.5 bar and then collected in a stainless-steel container filled with liquid nitrogen. After evaporation of the liquid nitrogen, the frozen droplets were transferred to a precooled vessel of 500 mL and freeze-dried for 48–72 h (−55 °C, vacuum of 10 Pa) to obtain SFD AHc dry powders (FD-1B-50 Vacuum Freeze-drying Systems, Biocool, Beijing)^35,53^. AHc was dissolved in PBS to obtain the AHc liquid vaccine (0.4 mg/mL); AHc dry powder was reconstituted in water at a ratio of 10:1 to obtain the AHc reconstituted powder vaccine.

### Characterization of inhalable AHc vaccines

The AHc dry powder yield was defined as the ratio of the weight of the sample after collection to the initial total dry mass. And the yield in % (w/w) was calculated (*n* = 3). The values were reported as mean ± SEM^54^. The AHc dry powder was reconstituted in deionized water and analyzed its stability by SDS-PAGE and western blot (using polyclonal antibodies collected from immunized mice)^55^.

The AHc particles between 0.4 and 10 μm were measured by using Malvern Zetasizer Nano S90 (Malvern Instruments Ltd, USA)^56^. The AHc dry powder was dispersed in anhydrous ethanol for sonication using probe and stabilized for 30 min. The MMAD of AHc aerosol particles was measured by a TSI APS spectrometer 3321 (TSI Inc, USA). For this process, the bio-aerosol generator was used to generate aerosols of AHc dry powder in a bio-settlement Cabinet (Huironghe Company, Beijing, China), and the TSI Model 3321 was used to measure the MMAD of aerosol AHc dry powders^57,58^.

TGA was used to determine the moisture content in the AHc dry powder samples. AHc dry powder (1 mg) was loaded into a thermal analyzer TA Instrument Q50 and heated from 30 to 250 °C (5 K/min) under nitrogen gas^54^. The morphology of the AHc dry powder was examined with a Hitachi S-3400N scanning electron microscope.

### Animal immunization

Mice (32 per group) were immunized three times at 21-day intervals^29,36^ by i.t. inoculation with 0.5 mg of AHc dry powder (i.t.-AHc, powder), 0.5 mg of AHc dry powder reconstituted in water (i.t.-AHc, powder reconstituted), or 20 μg of AHc liquid (AHc without SFD, i.t.-AHc, liquid) in PBS; or by s.c. injection with 0.5 mg of AHc dry powder (s.c.-AHc, powder), 0.5 mg AHc dry powder reconstituted in water (s.c.-AHc, powder reconstituted), or 20 μg of AHc liquid (AHc without SFD, s.c.-AHc, liquid). Mice (32 per group) immunized with (1) 0.5 mg of CpG powder by i.t. inoculation (i.t.-CpG, powder), (2) 0.5 mg of CpG dry powder reconstituted in water by i.t. or by s.c. (i.t.-CpG, powder reconstituted or s.c.-CpG, powder reconstituted), or (3) 20 μg of CpG in PBS by i.t. or by s.c. (i.t.-CpG, liquid or s.c.-CpG, liquid) served as negative controls. Mice immunized with 50 μL of PBS by the i.t. or s.c. route (i.t.-PBS or s.c.-PBS) served as blank controls. The immunization groups are shown in Table 2.

### Determination of AHc-specific antibody levels by ELISA

Eight mice per group were used to collect blood samples from the retro-orbital sinus 21 days after each immunization. Another four mice per group were euthanized to collect BAL 21 days after each immunization. For ELISA protocols, 96-well plates (Costar, USA) were coated with an optimal concentration of AHc (100 μL of 3 μg/mL) in PBS overnight at 4 °C. After the plates were blocked with 1% bovine serum albumin at 37 °C for 2 h, 100 μL of the diluted serum or BAL was added, and the plates were incubated at 37 °C for 45 min. Next, the plates were washed and then incubated with 100 μL (1:10,000 dilution) of horseradish peroxidase-conjugated goat anti-mouse IgG, IgG1, IgG2a, or IgA (Abcam, Cambridge, MA) at 37 °C for 45 min. The antibody titers to AHc were detected by a TMB substrate kit. Results were expressed as ODs measured at 450 nm by a Multiskan Mk3 microplate reader (Thermo Scientific, USA).

### Determination of cytokine secretion by T cells

Three mice per group were euthanized 63 dppi, and total mononuclear cells were isolated from spleens and suspended (1 × 10^7^/mL) in DMEM basic medium (Gibco, Shanghai, China) containing 10% (v/v) fetal bovine serum (Gibco, Australia) and 2% penicillin–streptomycin (Gibco, Grand Island, USA) in a 96-well ELISPOT plate (Mabtech, Nacka Strand, Sweden). Then 20 μg/mL AHc, 2.5 μg/mL Concanavalin A (ConA, positive control, Sigma-Aldrich, Germany), or cell culture medium (negative control) was added to triplicate wells, which were incubated for 40 h at 37 °C under 5% CO_2_. IFN-γ or IL-4 levels were measured by ELISPOT assays^21,29,59,60^.

### BoNT/A aerosol challenge

At 63 dppi, ten mice per group were challenged i.t. with either 10,000× LD_50_ or 30,000× LD_50_ BoNT/A complex in 50 μL of PBS. Mice were monitored hourly for the first 6 h and then daily for paralysis and death for 14 days. Mice were euthanized when signs (breathing difficulties and lack of mobility, etc) of morbidity were observed.

### BAL neutralization assay

Undiluted BAL of each group was incubated with 20× LD_50_ or 100× LD_50_ BoNT/A complex for i.t. challenge, and 100× LD_50_ or 500× LD_50_ BoNT/A complex for i.p. challenge, at a ratio of 1:1 in 0.2% gelatin/PBS for 30 min at room temperature^8,21^. The solution mixture was then injected into mice via the i.p. or i.t. route, and mice were monitored hourly for the first 6 h and then daily for paralysis and death for 7 days. Mice were euthanized when signs (breathing difficulties and lack of mobility, etc) of morbidity were observed.

### Histopathology

Part of lung, liver, and spleen of mice at 21 days after each immunization was collected and immediately fixed in formalin. The pathological changes in the hematoxylin–eosin-stained tissue sections were observed with light microscopy.

### Statistics

The data are expressed as mean ± SEM. All statistical analyses were performed using SAS statistical software (version 9.1, SAS Institute Inc., Cary, NC) or GraphPad Prism. The differences in the levels of antibodies among all groups of mice were assayed by two-way ANOVA, followed by LSD analysis or Tukey’s test. IFN-γ levels were compared by one-way ANOVA, followed by LSD analysis. The animal survival rate was analyzed by Kaplan–Meier survival estimates. Comparisons were considered significantly different if *P* < 0.05.

### Reporting summary

Further information on research design is available in the Nature Research Reporting Summary linked to this article.

## Supplementary information

Supplementary information

REPORTING SUMMARY

## Data Availability

All the relevant data are presented in this paper or within the Supplementary Information.
