# Supplementary material for: Intratracheal inoculation of AHc vaccine induces protection against aerosolized botulinum neurotoxin A challenge in mice
Source: NPJ Vaccines. 2021 Jun 22;6:87. doi: 10.1038/s41541-021-00349-w (PMC8219734; doi:10.1038/s41541-021-00349-w)
Supplement: Supplementary file 1 — Supplementary information [file 41541_2021_349_MOESM1_ESM.pdf]

Supplementary Figures

Supplementary Figure 1. The full, un-cropped images of Fig.1a.

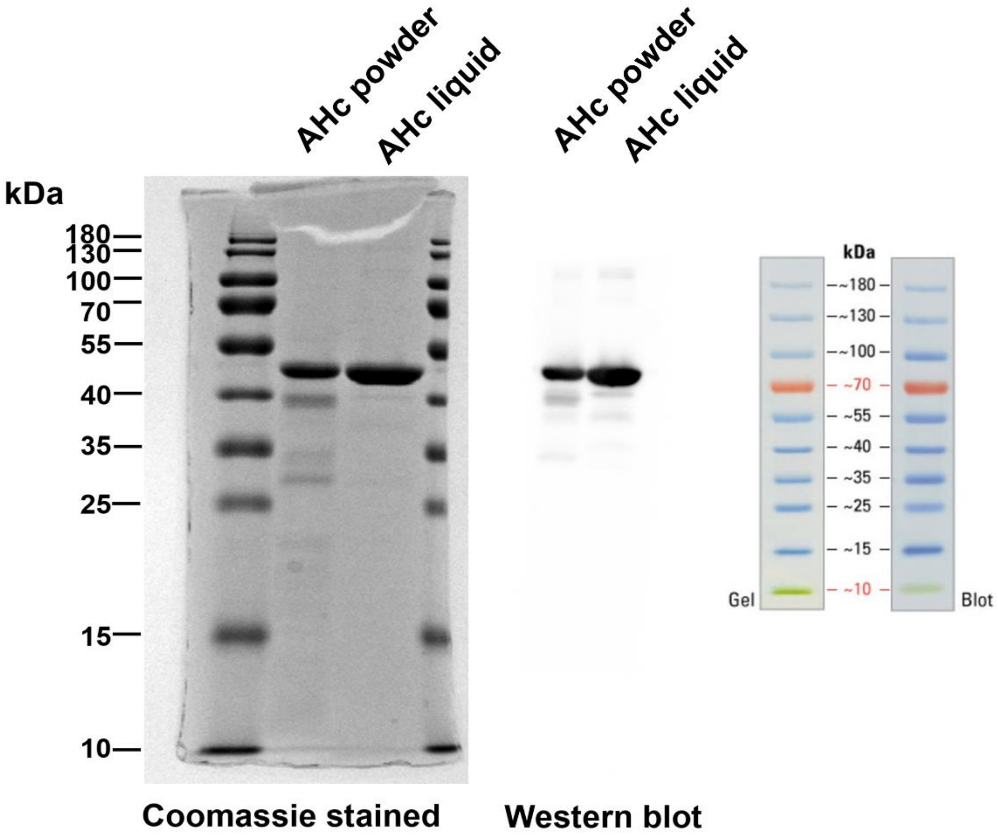

Supplementary Figure 2. IFN-γ ELISPOT-based quantification of peptide-specific IFN-γ-producing T cells in AHc-immunized mice. T cells were individually isolated from the spleens of three mice from each group at 63 dppi and stimulated with AHc for 40 h, and then the levels of IFN-γ were determined by ELISPOT. Data are presented as mean ± SEM of n = 3 mice per group. Statistical differences were calculated by one-way ANOVA, followed by LSD analysis. *$P < 0.05$.

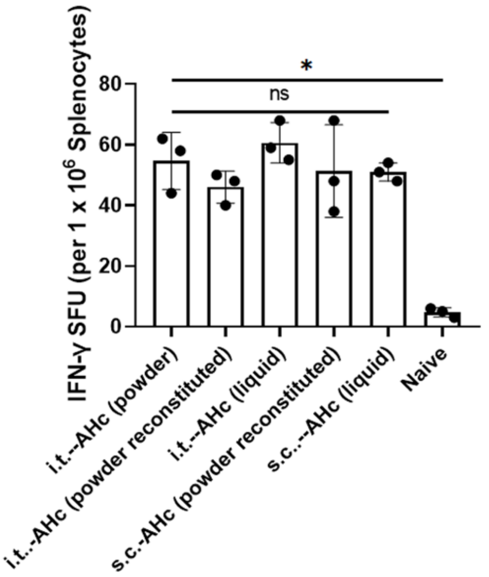

Supplementary Figure 3. Lethality of mice exposed to BoNT/A. (a) i.t. route, (b) i.p. route.

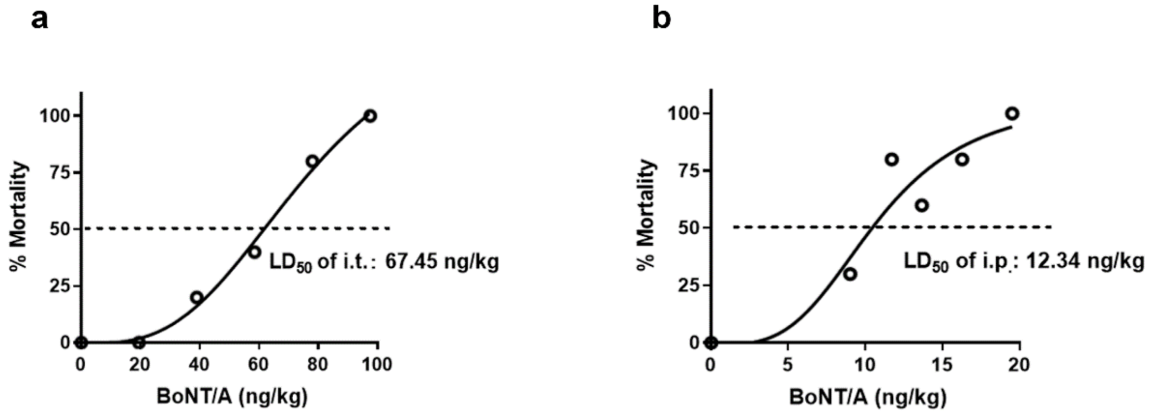

Supplementary Figure 4. Body weight changes of mice challenged with BoNT/A via i.t. route and i.p. route. 5 groups of mice (10 in each group) were i.t. route challenged with (a) 10,000×i.t. LD_{50} or (b) 30,000×i.t. LD_{50} BoNT/A. Weights and survival status of all the mice were recorded 14 days post challenge. Each point represents the change in of the mice as the arithmetic mean ± SEM in each group.

a

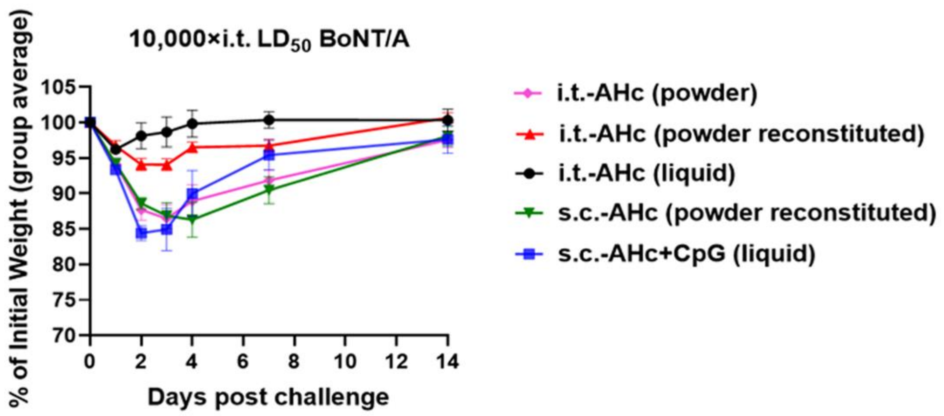

b

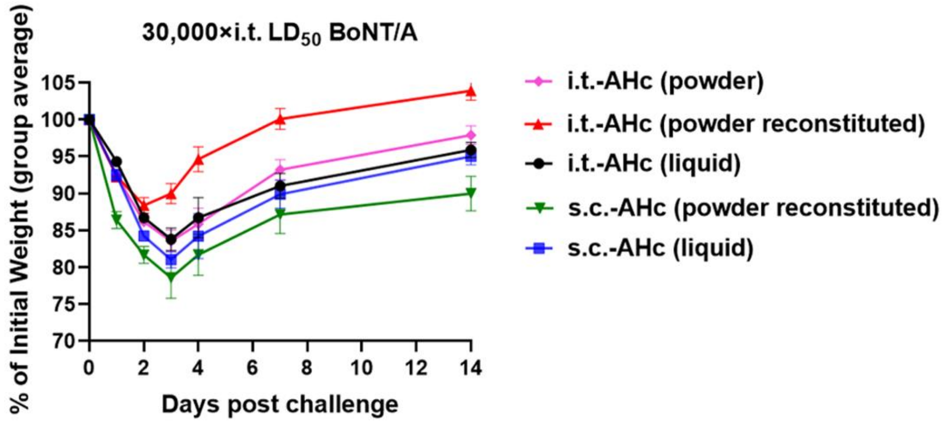

Supplementary Figure 5. Pathological changes of mice on 21 days after each immunization. 4 mice per group were euthanized on 21 days after each immunization and part of their lung and spleen were collected. The (a) lung, (b) spleen and (c) liver were treated by conventional histopathological methods and observed in the HE stained sections by a light microscopy (original magnifications 400, bar=100 μm). (d) The pathological scores of lung tissue. (e) The pathological scores of spleen tissue. (f) The pathological scores of liver tissue. Tissue sections were evaluated by a trained pathologist according to the following scores: 0, no pathological lesions; 1, minimal; 2, mild; 3, moderate; 4, severe. The experiments were performed twice independently with

similar results. Data were expressed as the mean ± SD (n = 4) that collected from one representative experiment. * $P < 0.05$.

a

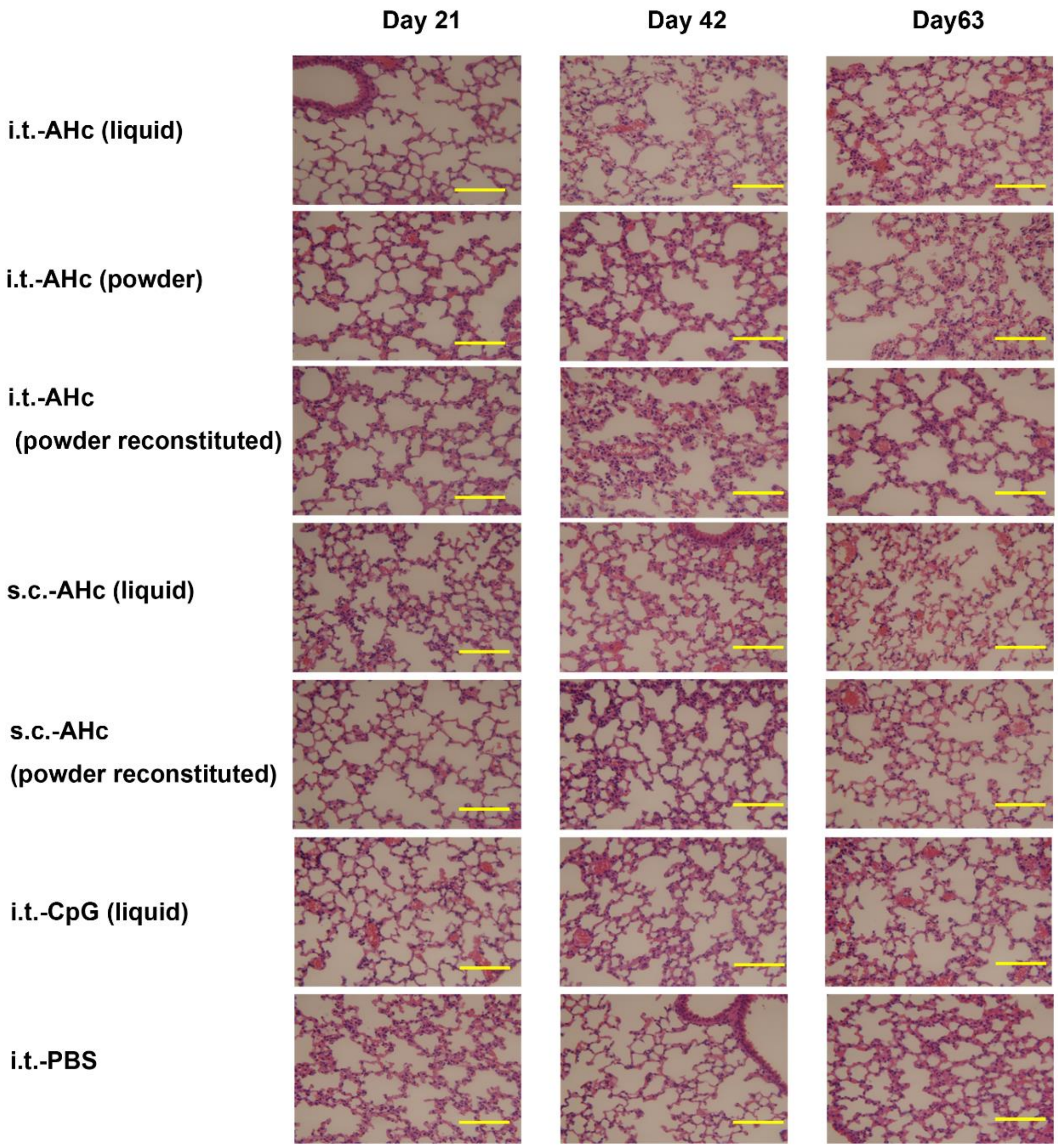

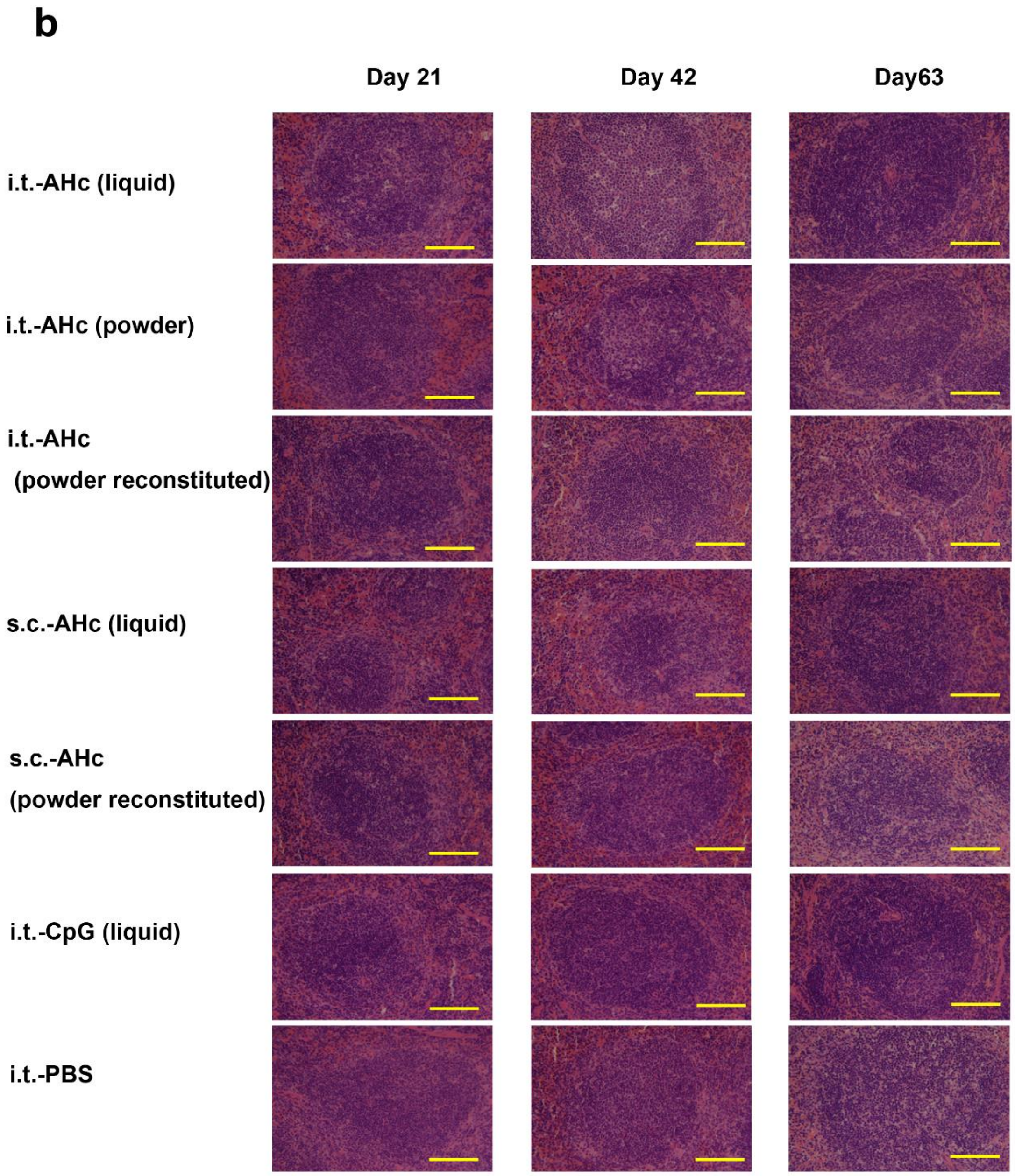

b
Day 21
Day 42
Day63
i.t.-AHc (liquid)
i.t.-AHc (powder)
i.t.-AHc
(powder reconstituted)
s.c.-AHc (liquid)
s.c.-AHc
(powder reconstituted)
i.t.-CpG (liquid)
i.t.-PBS

c

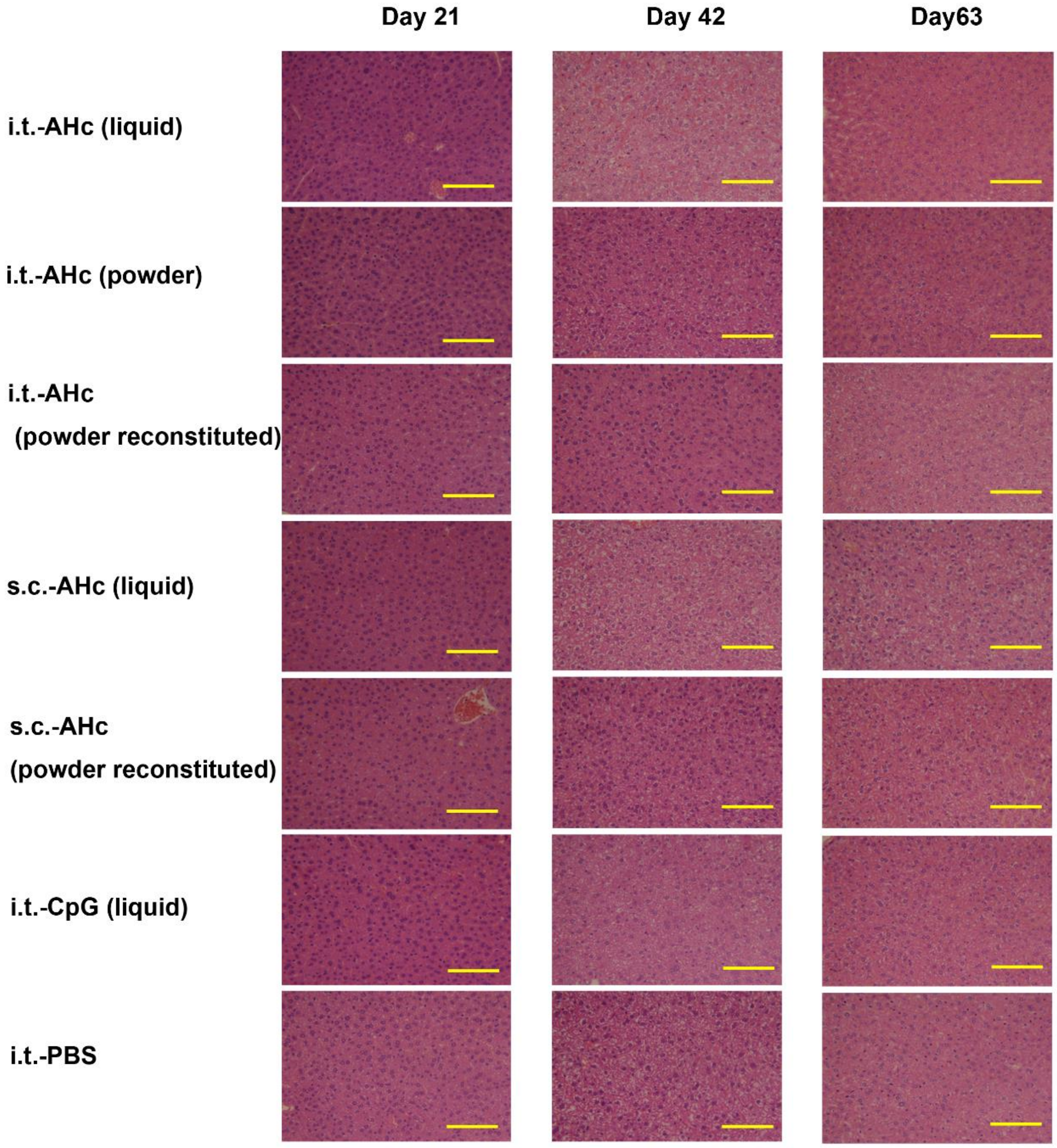
Day 21
Day 42
Day63
i.t.-AHc (liquid)
i.t.-AHc (powder)
i.t.-AHc
(powder reconstituted)
s.c.-AHc (liquid)
s.c.-AHc
(powder reconstituted)
i.t.-CpG (liquid)
i.t.-PBS

d

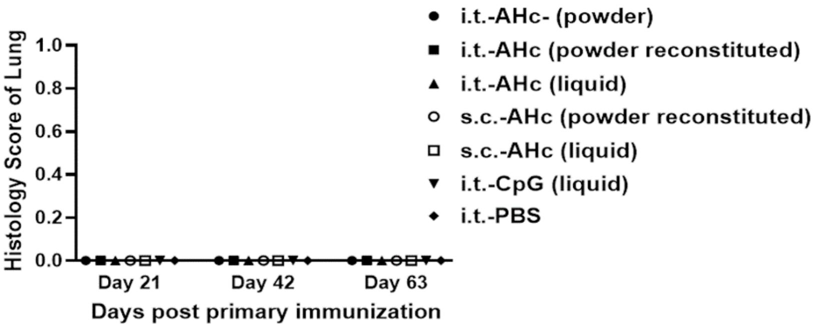
Histology Score of Lung
1.0
0.8
0.6
0.4
0.2
0.0
Day 21
Day 42
Day 63
Days post primary immunization
i.t.-AHc- (powder)
i.t.-AHc (powder reconstituted)
i.t.-AHc (liquid)
s.c.-AHc (powder reconstituted)
s.c.-AHc (liquid)
i.t.-CpG (liquid)
i.t.-PBS

e

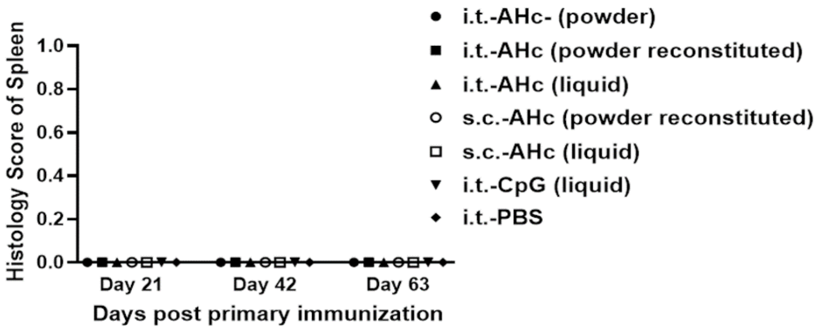
Histology Score of Spleen
1.0
0.8
0.6
0.4
0.2
0.0
Day 21
Day 42
Day 63
Days post primary immunization
i.t.-AHc- (powder)
i.t.-AHc (powder reconstituted)
i.t.-AHc (liquid)
s.c.-AHc (powder reconstituted)
s.c.-AHc (liquid)
i.t.-CpG (liquid)
i.t.-PBS

f

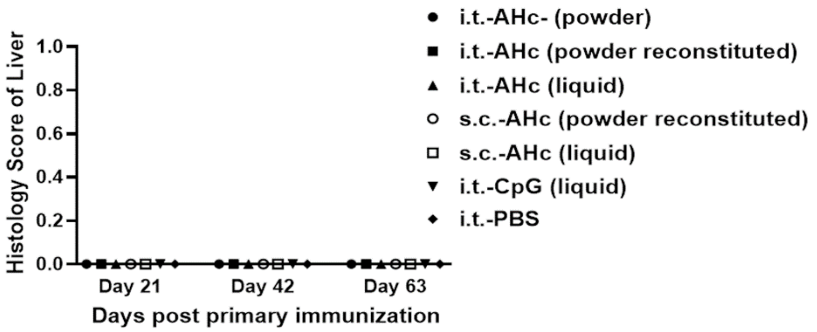
Histology Score of Liver
1.0
0.8
0.6
0.4
0.2
0.0
Day 21
Day 42
Day 63
Days post primary immunization
i.t.-AHc- (powder)
i.t.-AHc (powder reconstituted)
i.t.-AHc (liquid)
s.c.-AHc (powder reconstituted)
s.c.-AHc (liquid)
i.t.-CpG (liquid)
i.t.-PBS
